# Mining and analysis of microsatellites in human coronavirus genomes using the in-house built Java pipeline

**DOI:** 10.5808/gi.20033

**Published:** 2022-09-30

**Authors:** Umang, Pawan Kumar Bharti, Akhtar Husain

**Affiliations:** 1 School of Computer Science, Shri Venkateshwara University, Gajraula 244236, Uttar Pradesh, India; 2 Department of Computer Science and IT, MJP Rohilkhand University, Bareilly 243006, Uttar Pradesh, India

**Keywords:** compound simple sequence repeats, human coronavirus, MISA, perfect simple sequence repeats, primer design, relative abundance, relative density

## Abstract

Microsatellites or simple sequence repeats are motifs of 1 to 6 nucleotides in length present in both coding and non-coding regions of DNA. These are found widely distributed in the whole genome of prokaryotes, eukaryotes, bacteria, and viruses and are used as molecular markers in studying DNA variations, gene regulation, genetic diversity and evolutionary studies, etc. However, *in vitro* microsatellite identification proves to be time-consuming and expensive. Therefore, the present research has been focused on using an in-house built java pipeline to identify, analyse, design primers and find related statistics of perfect and compound microsatellites in the seven complete genome sequences of coronavirus, including the genome of coronavirus disease 2019, where the host is *Homo sapiens*. Based on search criteria among seven genomic sequences, it was revealed that the total number of perfect simple sequence repeats (SSRs) found to be in the range of 76 to 118 and compound SSRs from 01 to10, thus reflecting the low conversion of perfect simple sequence to compound repeats. Furthermore, the incidence of SSRs was insignificant but positively correlated with genome size (R^2^ = 0.45, p > 0.05), with simple sequence repeats relative abundance (R^2^ = 0.18, p > 0.05) and relative density (R^2^ = 0.23, p > 0.05). Dinucleotide repeats were the most abundant in the coding region of the genome, followed by tri, mono, and tetra. This comparative study would help us understand the evolutionary relationship, genetic diversity, and hypervariability in minimal time and cost.

## Introduction

Coronaviruses, first identified in the mid-1960s, are a group of RNA viruses that causes respiratory illness in mammals and birds; these constitute the subfamily *Orthocoronavirinae* in the family *Coronaviridae* [1,2]. They have club-shaped spikes projecting from their surface, so the name has been derived from the Latin word "Corona", meaning crown. The term was first coined by June Almeida and David Tyrrell, who first observed and studied human coronaviruses. Coronavirus was accepted as a genus name in 1971 [3]. As the number of new species increased, the genus was split into four genera: Alphacoronavirus, Betacoronavirus, Deltacoronavirus, and Gammacoronavirus. The seven commonly found coronaviruses, where the host is *Homo sapiens* are: 229E (alpha coronavirus), NL63 (alpha coronavirus), OC43 (beta coronavirus), and HKU1 (beta coronavirus); the rest uncommon viruses found are MERS-CoV (Middle East respiratory syndrome coronavirus; the beta coronavirus that caused Middle East respiratory syndrome or MERS), SARS-CoV (severe acute respiratory syndrome coronavirus; the beta coronavirus that caused severe acute respiratory syndrome or SARS), and SARS-CoV-2 (severe acute respiratory syndrome coronavirus 2; the novel coronavirus that causes coronavirus disease 2019 or coronavirus disease 2019 [COVID-19]). People commonly get infected with human coronaviruses 229E, NL63, OC43, and HKU1. Although significant genetic diversity of coronaviruses was detected from Shenzhen in Mainland China and Hong Kong ports, all the strains had a high homology compared with the published strains; several novel mutations, including nucleotides substitution and the insertion of the spike of the glycoprotein on the viral surface, were discovered [4]. Sometimes coronaviruses that infect animals can evolve and make people sick and become a new human coronavirus. Three recent examples of these are SARS-CoV-2, SARS-CoV, and MERS-CoV. SARS-CoV-2 is a strain of coronavirus that causes COVID-19 and is responsible for respiratory illness in human beings; this was first identified in Wuhan city of, China, in January 2020 (NCBI GenBank No. MN908947.3). Coronavirus contains a positive-sense, single-stranded RNA genome; their genome size ranges from approximately 26 to 32 kb. The treatment of coronavirus is symptomatic; the transmission can be reduced by practising hygienic measures and getting the vaccination. Currently, three major approaches are being followed for designing vaccines: the whole microbe approach, the subunit approach, and the genetic approach. The mRNA and viral vector vaccines were rapidly developed using the genetic and whole microbe approaches. Also, at least nine different technology platforms are under research and development to design an effective vaccine against COVID-19.

The viral genome research will contribute to understanding and solving numerous problems, including their origin, evolution, infection mechanism, disease treatment, etc. [5]. The origin and evolution of viruses can be better understood by investigating them at the molecular level [6-9]. Accumulation of transposable elements [10,11] and tandem repeats [12] are considered for changes in genome size. Ninety-two genome sequences of severe acute respiratory syndrome coronavirus 2 have been uncovered with the SARS-CoV-2 reference genome (NC_045512.2) [13]. The hypervariability and the hotspots of mutations in coronavirus genome sequences can be discovered by studying simple sequence repeats (SSRs).

"Microsatellites" or SSRs [14] are short tandem repeats (motifs) of lengths 1–6 nucleotides [15] and are found in the genomes of both prokaryotes and eukaryotes [16]. SSRs can be categorized as perfect [without interruptions, or we can say a continuous repeat of a single motif; (AGA) _15_], imperfect [with interruptions by non-repeat nucleotide or with a base pair disruption between repeats; (AGA) _7_ A (AGA) _8_] and compound [two or more SSRs are found adjacent to one another; (GTG) _8_ (AT) _16_] [17], also known as compound simple sequence repeats (cSSR). For a microsatellite to be categorized as a compound, the maximum permissible distance between two adjacent microsatellites is known as dMAX [18]. The dMAX value can be set only from 0 to 50 for IMEx [19]. These are present in the genome's coding and non-coding regions [20,21]. The SSRs found in the coding region affect gene activation, resulting in protein expression and lesser polymorphism in the coding part [22]. SSRs present in the non-coding area affects gene regulation [23]. These repeats may be generated due to the slippage mechanism during replication [24]. These microsatellites promote the development of markers widely used by researchers in DNA‐based genetic analyses for the past 25 years, which show locus speciﬁcity, high reproducibility, co-dominance inheritance and hypervariability [25]. The ﬂanking sequences of SSRs help select polymerase chain reaction primers that amplify the repeat sequence [26]. SSRs are essential in studying genetic variation, gene tagging, linkage mapping [27-29], and evolutionary studies [30]. Many researchers have reported the involvement of SSRs in transcription, translation, regulation of promoters [31,32] and certain neurodegenerative diseases [33].

Due to the importance of microsatellite applications in genomic research, various studies have been made to identify and characterise them in the laboratory. However, developing microsatellite markers *in vitro* is intensive and time-consuming [34]. The increasing availability of next-generation sequencing tools and genome sequences of various organisms in biological databases are providing a simple, fast and inexpensive way for *in silico* mining of SSRs [35].

In the present study, seven complete genome sequences of human coronavirus were mined and analyzed for perfect and compound SSRs occurrence and abundance by an in-house Java pipeline. Similar strains with sequence identity above 99.97% from different regions indicative of very recent emergence were not considered.

Because of the pandemic outbreak and loss to human health and the economy, it is essential to explore the virus genome to study and analyze the SSRs pattern to help establish an evolutionary relationship, genetic diversity, and genetic similarity/dissimilarity. Furthermore, analysis of perfect repeats would also help study the polymorphic nature and suitability for marker developments by using computing methods in less time and at no cost within the two genera Alphacoronvirus and Betacoronavirus.

## Methods

### Input files

Complete genome sequences in Genbank and FASTA format were downloaded from NCBI GenBank (https://www.ncbi.nlm.nih.gov/genbank/) with accession numbers (human coronavirus 229E: NC_002645.1, human coronavirus NL63: NC_005831.2, human coronavirus OC43: NC_006213.1, human coronavirus HKU1: NC_006577.2, SARS coronavirus: NC_004718.3, MERS-CoV/THA/CU/17_06_2015: KT225476.2, and severe_acute_respiratory_syndrome_coronavirus_2_isolate_Wuhan-Hu-1: NC_045512.2).

### The technology used for identification and analysis

**Fig. 1.**
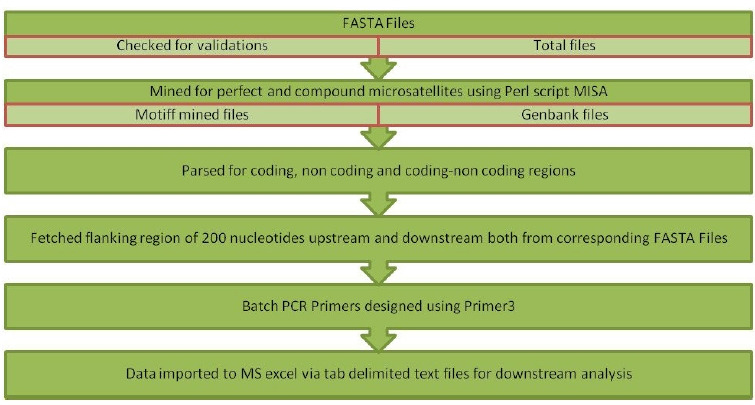
Graphical user interface showing resetting repeat numbers and saving them to the configuration file. Upload, FASTA files button allows uploading files. In addition, the option to upload a GenBank file is available for fetching other genomic features. Mine simple sequence repeats button displays the alert box showing batch submission and processing of FASTA and GenBank files for mining simple sequence repeats and designing primers by fetching flanking regions.

**Fig. 2.**
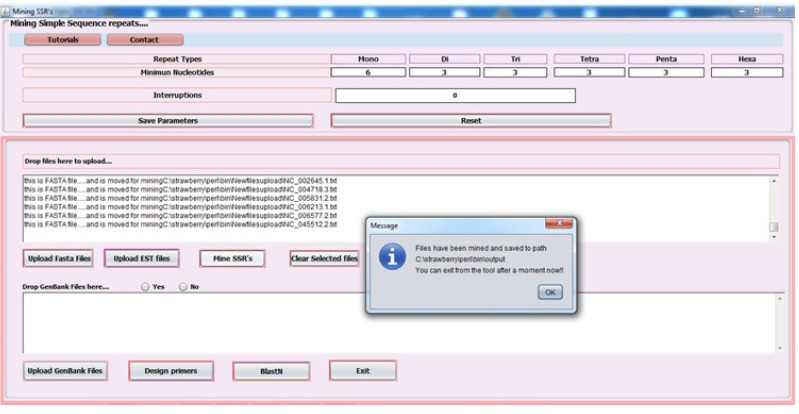
Workflow demonstration of in-built Java pipeline using misa.pl Perl script and Primer3 software with customized parameters.

Batch processing of Input files was performed through the in-house standalone tool with an interactive, user-friendly graphical user interface designed using Java Net Beans IDE 8.0.2; it is a robust and platform-independent technology. Strawberry Perl version 5.20.1.1 was used for the implementation of the Perl script. Misa.ini, a configuration file, was used to set the number of interruptions and repeat size. In this study, parameters for repeat numbers were set as 6, 3, 3, 3, 3, and 3 for mono to hexanucleotides repeats, respectively, with zero interruptions.Misa.pl [36], a Perl script that was used for mining perfect SSR and cSSR. The algorithm has been written using Java programming language that performs a call to misa.ini, misa.pl and Primer3 software [37] with default parameters (Fig. 1). The flanking regions of 200 nucleotides were fetched in the pipeline to design batch primers for the identified microsatellites. Outputs written in tab-delimited text files were imported into MS Excel 2007 for further downstream analysis. The workflow implemented via pipeline is demonstrated in Fig. 2.

Compound microsatellites extraction was performed with Imperfect Microsatellite Extractor (IMEx) software with the same number of repeat sizes as for perfect SSRs but with dMAX 10.

## Results

### Identification and distribution perfect SSR and cSSR in the genome sequences under study

**Fig. 3.**
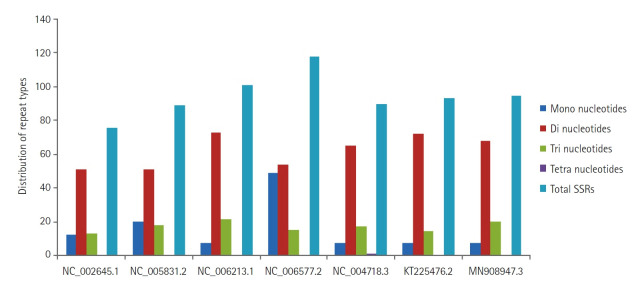
The distribution of repeat types from mono to tetranucleotides in coronavirus genome sequences with accession number’s mentioned on the horizontal axis. SSR, simple sequence repeat.

Six hundred sixty-two SSRs were identified within the genome sequences under study. Perfect repeats ranged from 76 (human coronavirus 229E) to 118 (human coronavirus HKU1). In the present study, cSSR were extracted with dMAX set at value 10, and cSSR extracted were found to be in the range from 01 (SARS and MERS coronavirus) to 10 (human coronavirus HKU1), thus reflecting the low conversion of SSRs to cSSR. Of the total SSRs identified, dinucleotide repeat motifs (51 to 73) were predominant, followed by trinucleotide repeat motifs (13 to 21), mononucleotide repeat motifs (07 to 49) and only rare tetra-nucleotide repeat motifs were observed in SARS coronavirus (Fig. 3). Penta and hexanucleotide repeats were found missing. For the mono, di and trinucleotide repeat motifs, the frequency is as high as 99.99%. The most abundant mononucleotide motif was T and A, accounting for 100% of mononucleotide motif repeats. In dinucleotide repeats, the most frequent motif was TG and GT, followed by AT and TA. Later one is represented with the approximate distribution of 12%‒15%, which is an established platform for SSRs mutability. A high incidence of AT/TA may lead to an unstable genome sequence. Of the trinucleotide repeats, TGT/TTG was observed to be the most abundant. The presence of different repeat motifs revealed that the number of SSRs with shorter length was much higher than that with longer motifs (Fig. 3).

### Relative abundance and relative density of SSRs and cSSR

Values of relative abundance and relative density allow parallel comparison of different size genome sequences. Relative abundance is calculated by dividing the total number of SSRs by kilobase pair (kb) sequences. Relative density is calculated by dividing the total SSRs sequence by kb of sequences. Relative abundance ranged from 2.78 in human coronavirus 229E to 3.94 in human coronavirus HKU1, while in cSSR, it was found maximum at 0.33 in human coronavirus HKU1. Relative density was found to be in the range of 19.54 (human coronavirus 229E) to 26.23 (human coronavirus HKU1), and in cSSRs, it was lowest in MERS and highest in human coronavirus HKU1 (Table 1).

### Comparative distribution across coding and non-coding regions

**Fig. 4.**
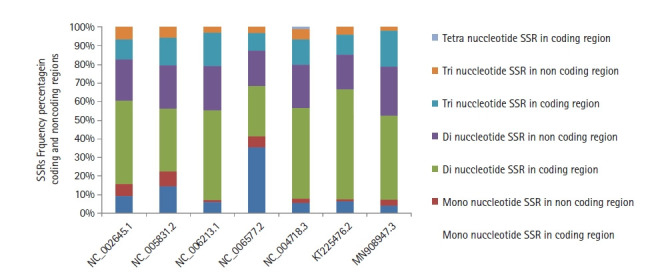
The distribution of mono, di, tri, and tetranucleotides simple sequence repeats (SSRs) frequency in the coding and non-coding regions of coronaviruses genome sequences with accession numbers mentioned on the horizontal axis.

The distribution of SSRs motifs among coding/non-coding regions in the human coronavirus genomes under study revealed a high incidence of 64.47% (human coronavirus 229E) to 72.0% (human coronavirus HKU1) of repeats within coding regions as compared to the non-coding areas. In addition, dinucleotide repeats in the coding region were predominantly followed by tri and mono (Fig. 4). Similarly, dinucleotides were also predominant in the non-coding areas, followed by tri and mono repeats (Supplementary Fig. 1).

### Statistical analysis

The correlation coefficient was tested between genome size/GC content to perfect SSRs number, relative abundance, relative density, cSSR number, cSSR relative abundance, cSSR relative density, and cSSR percentage. The incidence of SSRs was insignificant but positively correlated with genome size (R^2^ = 0.45, p > 0.05). Similarly, SSRs relative abundance R^2^ = 0.18, p > 0.05, SSRs relative density R^2^ = 0.23, and p > 0.05 were found to be insignificant but positively correlated with genome size; these results are in line with the study performed in deciphering the SSRs incidences across viral members of *Coronaviridae* family [38]. The cSSR number (R^2^ = 0.008, p > 0.05), cSSR relative abundance (R^2^ = 0.004, p > 0.05), cSSR relative density (R^2^ = 0.002, p > 0.05) and cSSR % (R^2^ = 0.004, p > 0.05) were found to be insignificant but positively correlated with genome size. Similarly, the Incidence of SSRs was found to be negatively correlated with GC content (R^2^ = 0.35, p < 0.05), also SSR relative abundance (R^2^ = 0.59, p < 0.05) and SSR relative density (R^2^ = 0.60, p < 0.05) were negatively correlated. The cSSR number (R^2^ = 0.85, p < 0.05), cSSR relative abundance (R^2^ = 0.87, p < 0.05), cSSR relative density (R^2^ = 0.83, p < 0.05), and cSSR % (R^2^ = 0.90, p < 0.05) were negatively correlated to GC content.

### Primer design for perfect repeats

**Fig. 5.**
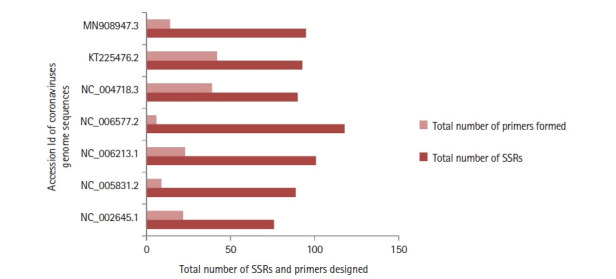
Graph depicting the number of primers formed compared to the number of simple sequence repeats (SSRs) in the corresponding genome sequence mentioned with accession numbers.

Among all seven genomic sequences, primer pairs were designed by fetching 200 nucleotides flanking regions both up and downstream of a motif by using custom settings as SEQUENCE_TEMPLATE=200[motif]200,SEQUENCE_TARGET=201,12,PRIMER_TASK= pick_detection_primers,PRIMER_PICK_LEFT_PRIMER=1,PRIMER_PICK_INTERNAL_OLIGO=1,PRIMER_PICK_RIGHT_PRIMER=1,PRIMER_OPT_SIZE=18,PRIMER_MIN_SIZE=15,PRIMER_MAX_SIZE=21,PRIMER_MAX_NS_ACCEPTED=1,PRIMER_PRODUCT_SIZE_RANGE=75-100,P3_FILE_FLAG=1,SEQUENCE_INTERNAL_EXCLUDED_REGION=201, 12, PRIMER_EXPLAIN_FLAG=1. Accession number-wise, the number of motifs, the number of primers formed, and the total percentage are mentioned in Table 2. Motifs along with corresponding start-end position, length, coding-non coding region, forward/reverse primer pairs, primers length, GC content, product size, melting temperature (TM) and stability were recorded. A record of microsatellites for which primers were not formed due to insufficient flanking regions or poor melting temperature was also maintained. In Fig. 5, bar graph is displayed showing the number of primers formed compared to the number of simple sequences repeats in the corresponding genome sequence mentioned with corresponding accession numbers.

## Discussion

The incidence of SSRs and cSSR distribution exhibits a similar pattern as reported in earlier studies in genomes of the *Filoviridae* family [34]. Of the total SSRs identified, dinucleotide repeat motifs (51 to 73) were predominant as found in *Flavivirus* genomes, and Mycobacteriophage genomes of *the *Siphoviridae** family [35,36] may be unstable due to higher slippages rate [37]. The presence of poly (T/A) is in line with the prokaryotic and eukaryotic genomes having abundant poly (T/A) tracts [14,39]. Mononucleotide A was plentiful, and in plant viroids, it tends to form loops in secondary structure and a possibly higher number of repeats, making it more difficult to form stable base pairs [40]. The cSSR percentage increases with dMAX size nonlinearly; this conversion of SSRs to cSSR in approximate similar size genomes suggests a differential role of repeat sequences [39]. At least one cSSR in each human coronavirus genome may be responsible for variation and evolution [41]. Di and trinucleotide repeats were mainly present in the coding region [39,42]. As far as motif types and their distribution in the coding and non-coding area is concerned, the reference sequence of SARS-CoV-2 (accession No. NC_045512.2) is close to the SARS virus genome (accession No. NC_004718.3), levels of the genetic relationship were also suggested among Bat coronavirus RaTG13 and the prototype strain of SARS-CoV-2 [43].

SSRs are insignificantly but positively correlated to genome size; the longer the genome size, the greater the number of SSRs [5,12,44] and repeat length. The study of the relationship between genome size and tandem repeat length in CoV HKU1 strains, a beta coronavirus, also provides evidence of a similar pattern to our findings [45].

The Insignificant correlation between genome size to relative abundance and density has also been found in the case of *Escherichia coli* and human Immunodeficiency virus type 1 (HIV-1) genome. These results reflect a slight effect of genome size on the relative abundance and density of SSRs in viral genomes [5,46]. As observed, the negative correlation of GC content with SSRs, relative abundance, relative density, and cSSR was also reported [39,41,42].

A literature survey observed that limited research had been done on identifying and analysing microsatellites in virus genomes. The study conducted on eukaryotic and prokaryotic genome sequences, including in-depth analysis of *Flavivirus*, Dengue virus, HIV, plant viroids, Ebolavirus, *Filoviridae*, and *Siphoviridae* family genomes, revealed the role of genome size in accumulation of numbers and length of SSRs also to particular extent host are also found responsible for variances as they may participate in recombination and integration [47,48]. An increase in SSRs numbers may be due to the combination of partial sequences of the host during the infection [44]. All parameters under study were relevant and matched with previous research [5]. The maximum primers were designed in MERS-CoV/THA/CU/17_06_2015 with accession No. KT225476.2 followed by accession No. NC_004718.3 which is a SARS-CoV and least in human coronavirus HKU1 with accession No. NC_006577.2. Overall, in our study, it has been observed that HKU1 is showing a slightly different pattern in SSRs and cSSR abundance per kb and consequently in relative abundance, density and GC % content; such a pattern has also been highlighted in earlier studies in screening microsatellites in 55 *Coronaviridae* genomes [38], and it is among the top four strains found to be infecting human beings.

This study revealed the microsatellite identification, distribution, and analysis in seven genomic sequences of human coronavirus strains, including the reference sequence of SARS-CoV-2. From computational and statistical data, it was observed that the greater the genome size more is the SSRs number/length of repeats. The presence of a minimum of one compound SSRs, poly T and A mononucleotides, and abundant presence of AT/TA dinucleotides may be responsible for variation, instability, and evolution of the genome. It may contribute to understanding the genetic diversity and polymorphic nature of the genomes among alpha and beta-coronavirus genera. However, further study can elaborate on the mutable hotspots.

## Figures and Tables

**Table 1.** Genome-wide analysis results of perfect and compound simple sequence repeat from genome sequences under study, showing relative abundance and density variations

S. No.	Name	Accession No.	Genome size (bp)	GC (%)	SSR	RA	RD	cSSR	cRA	cRD	cSSR (%)
1	Human coronavirus 229E	NC_002645.1	27,317	38.3	76	2.78	19.54	3	0.1	1.86	3.94
2	Human coronavirus NL63	NC_005831.2	27,553	34.5	89	3.23	21.99	4	0.14	2.35	4.49
3	Human coronavirus OC43	NC_006213.1	30,741	36.8	101	3.28	22.93	4	0.13	1.91	3.96
4	Human coronavirus HKU1	NC_006577.2	29,926	32.1	118	3.94	26.23	10	0.33	5.94	8.47
5	SARS coronavirus	NC_004718.3	29,751	40.8	90	3.02	21.44	1	0.03	0.43	1.11
6	MERS-CoV/THA/CU/17_06_2015	KT225476.2	29,809	41.2	93	3.11	20.39	1	0.03	0.36	1.07
7	Severe_acute_respiratory_syndrome_coronavirus_2_isolate_Wuhan-Hu-1	MN908947.3/	29,903	38	95	3.17	22.53	3	0.1	2.4	3.15
		NC_045512.2									

GC (%), guanine-cytosine percentage; SSR, simple sequence repeats; RA, relative abundance; RD, relative density; cSSR, compound simple sequence repeats; cRA, the relative abundance of compound simple sequence repeats; cRD, the relative density of compound simple sequence repeats; cSSR (%), percentage occurrence of compound simple sequence repeats.

**Table 2.** The accession ID of coronavirus genome sequences, total numbers of SSRs, number of primers formed, and the percentage

S. No.	Accession ID	Total SSRs obtained	Primers formed	% Formed
1	NC_002645.1	76	22	28.9
2	NC_005831.2	89	9	10.1
3	NC_006213.1	101	23	22.8
4	NC_006577.2	118	6	5
5	NC_004718.3	90	39	43.3
6	KT225476.2	93	42	45.1
7	MN908947.3/	95	14	14.7
	NC_045512.2			

SSR, simple sequence repeat.

## Supplementary Materials

Supplementary Fig. 1. The detailed distribution of simple sequence repeat types among coding and non-coding regions in the human coronavirus genomes under study has been provided in supplementary files.
