# Supplementary material for: Mining and analysis of microsatellites in human coronavirus genomes using the in-house built Java pipeline
Source: Genomics Inform. 2022 Sep 30;20(3):e35. doi: 10.5808/gi.20033 (PMC9576472; doi:10.5808/gi.20033)
Supplement: Supplementary Fig. 1. [file gi-20033suppl1.pdf]

Motif types	NC_002645.1	NC_005831.2	NC_006213.1	NC_006577.2	NC_004718.3	KT225476.2	MN908947.3/	Total
Mono	12	20	7	49	7	7	7	109
Di	51	51	73	54	65	72	68	434
Tri	13	18	21	15	17	14	20	118
Tetra					1			1
Total SSR	76	89	101	118	90	93	95	662
								0

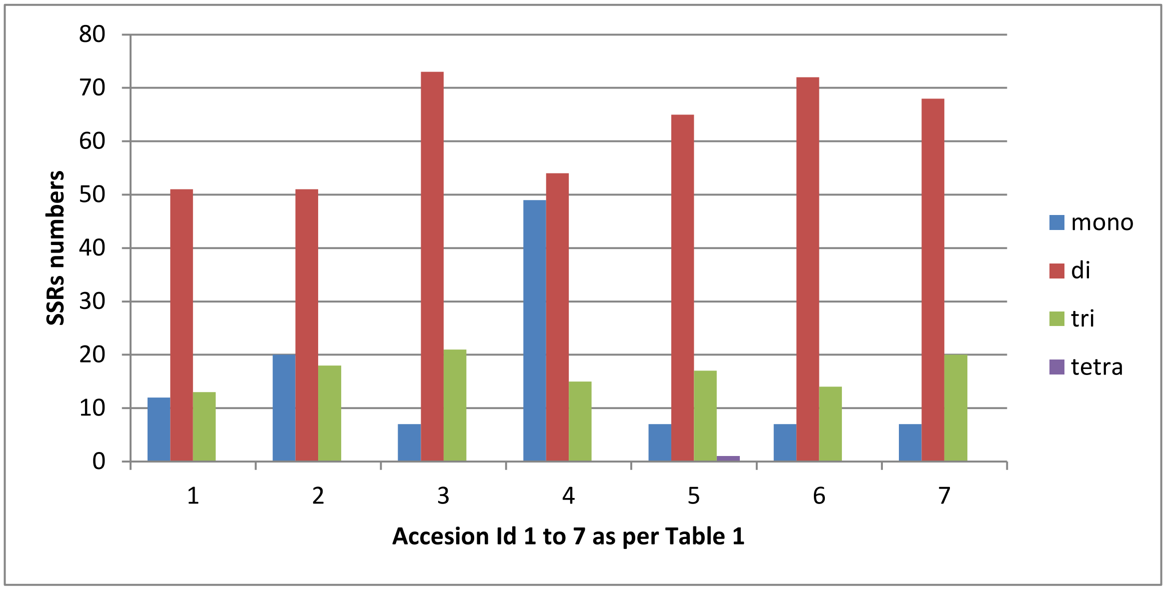

Motif types Information

Motif types	NC_002645 .1	NC_005831 .2	NC_006213 .1	NC_006577 .2	NC_004718 .3	KT225476 .2	MN908947. 3/	Total
Mono-coding	7	13	6	42	5	6	4	83
Mono-noncoding	5	7	1	7	2	1	3	26
Di coding	34	30	49	32	44	55	43	287
Di noncoding	17	21	24	22	21	17	25	147
Tri coding	8	13	18	11	12	10	18	90
Tri noncoding	5	5	3	4	5	4	2	28
Tetra coding	0	0	0	0	1	0	0	1
	76	89	101	118	90	93	95	662
	49	56	73	85	61	71	65	

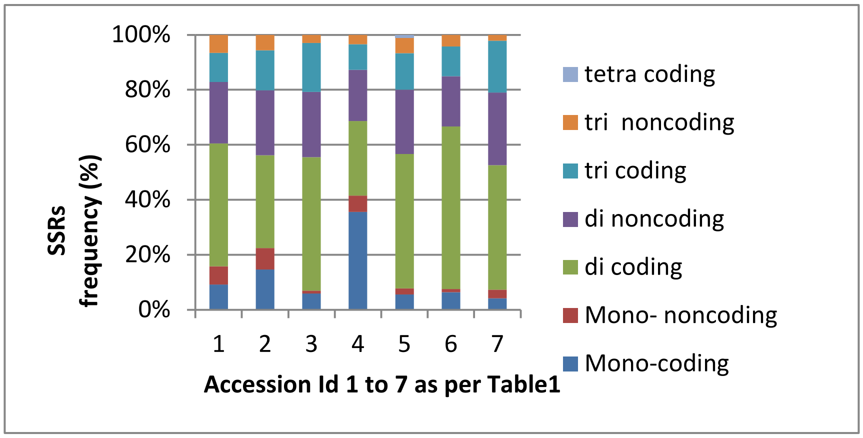

Supplementary Fig. 1. ?
